# Integrating CRISPR/Cas systems with programmable DNA nanostructures for delivery and beyond

**DOI:** 10.1016/j.isci.2022.104389

**Published:** 2022-05-11

**Authors:** Petteri Piskunen, Rosalind Latham, Christopher E. West, Matteo Castronovo, Veikko Linko

**Affiliations:** 1 Biohybrid Materials, Department of Bioproducts and Biosystems, Aalto University, P.O. Box 16100, 00076 Aalto, Finland; 2 Centre for Plant Sciences, University of Leeds, Leeds, UK; 3 School of Food Science and Nutrition, University of Leeds, Leeds, UK; 4 LIBER Center of Excellence, Aalto University School of Chemical Engineering, P.O. Box. 16100, 00076 Aalto, Finland

**Keywords:** Genetics, Nanotechnology, Nanostructure

## Abstract

Precise genome editing with CRISPR/Cas paves the way for many biochemical, biotechnological, and medical applications, and consequently, it may enable treatment of already known and still-to-be-found genetic diseases. Meanwhile, another rapidly emerging field—structural DNA nanotechnology—provides a customizable and modular platform for accurate positioning of nanoscopic materials, for e.g., biomedical uses. This addressability has just recently been applied in conjunction with the newly developed gene engineering tools to enable impactful, programmable nanotechnological applications. As of yet, self-assembled DNA nanostructures have been mainly employed to enhance and direct the delivery of CRISPR/Cas, but lately the groundwork has also been laid out for other intriguing and complex functions. These recent advances will be described in this perspective.

## Graphical abstract

**Figure:**
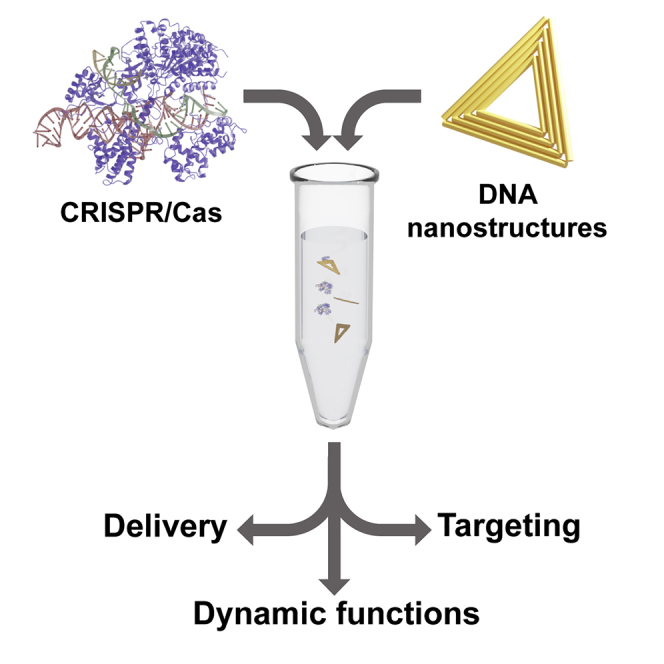

## Introduction

**Figure 1:**
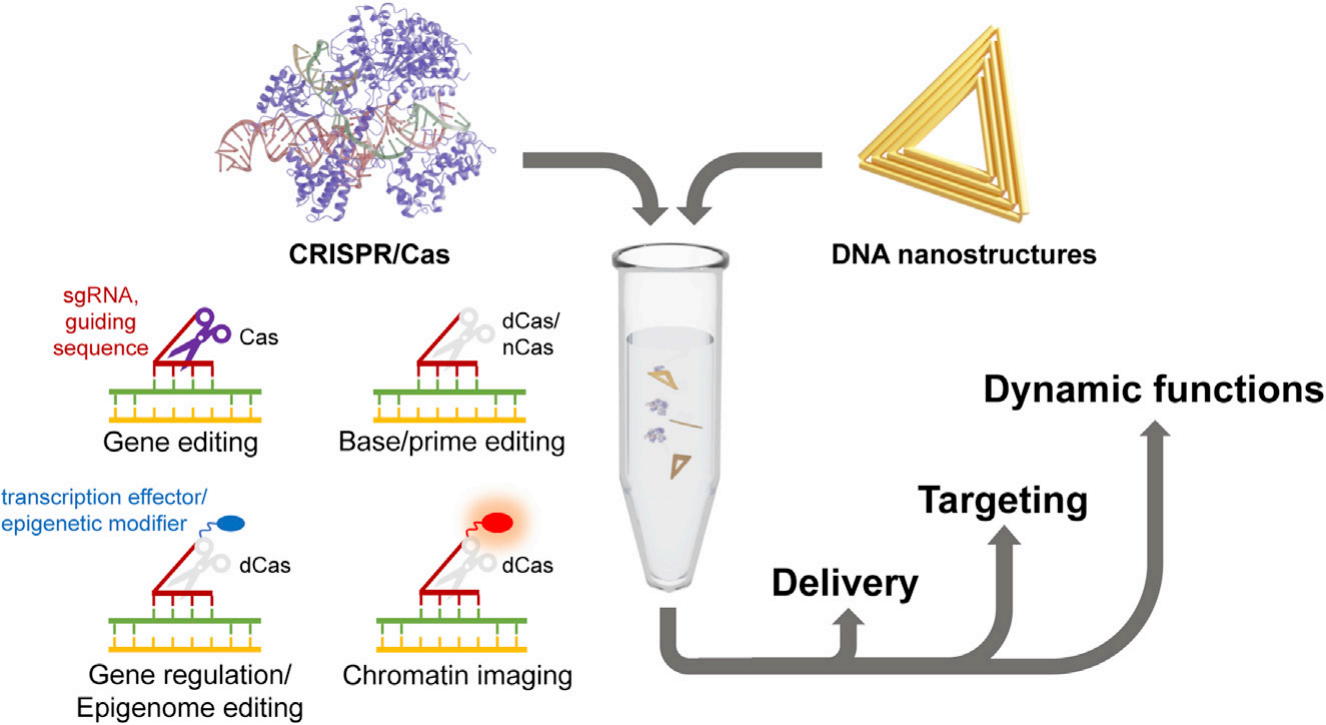
Diverse CRISPR/Cas functions combined with the DNA nanotechnology toolbox pave the way for new applications CRISPR/Cas tools for genetic editing (genes, bases, primes, and epigenomes), gene regulation and imaging can be integrated with programmable DNA nanostructures to facilitate various predefined functions. Here we discuss especially delivery and targeting but also dynamic operations that are coming increasingly into view. The CRISPR/Cas model is based on the entry CAS9_STAAU from the UniProt database (The UniProt Consortium, 2021).

The CRISPR-Cas system is a revolutionary molecular tool, enabling rapid, cheap, and targeted double-strand break (DSB) induction for varied applications. CRISPR (clustered regularly interspaced short palindromic repeats) describes the arrangement of genomic DNA motifs within bacteria and archaea that possess this form of adaptive immunity. CRISPR RNAs guide the CRISPR-associated-protein (Cas nuclease) to the target sequence (invading virus or plasmid) to cleave and destroy it through targeted DSB induction (Richter et al., 2012). The native CRISPR system was reengineered by Jennifer Doudna and Emmanuele Charpentier in 2012 (Nobel Prize in Chemistry in 2020) to simplify its application; rather than two guiding RNAs (crRNA and trRNA), a single guide RNA (sgRNA) directs the Cas9 nuclease to a programmable, complementary 20-nucleotide (nt) target sequence, whereupon a DSB is created (Jinek et al., 2012) (Figure 1). Similar results were also published from the Siksnys’ lab (Gasiunas et al., 2012).

Cutting of a target DNA sequence has many applications from analysis of gene function to generation of new disease models and gene therapies (Doudna and Charpentier, 2014). However, the standout application is that of genome engineering, where manipulation of DSB repair enables insertion of random or specific mutations or longer genetic sequences such as entire functional genes (Adli, 2018; Doudna, 2020). The great success of CRISPR-Cas has been in its easy reprogrammability of the target sequences, far more so than previous site-specific nucleases such as zinc-finger nucleases (ZFNs) or transcription activator-like effector nucleases (TALENs) (Doudna and Charpentier, 2014). Cas target sequences can be easily changed through alteration of the sgRNA sequence to target an alternative 20-nt protospacer in the genome versus complex protein engineering required for ZFN and TALENs. The range of potential target sites is also far greater for Cas nucleases than for TALENs and ZFNs, being only restricted by the occurrence of short protospacer adjacent motifs (PAMs) in the genome (e.g., Cas9: NGG, LbCas12a: TTTN). In contrast, ZFNs are made up of individual zinc finger proteins that each recognize a nucleotide triplet, but zinc fingers are only available for some CNN and TNN triplets (Wright et al., 2005), limiting the available target sites in the genome. Reprogramming TALEN target sites requires alteration of the amino acid (and therefore genetic) sequence of its constituent TAL effector DNA binding domains, which is hindered by the highly repetitive nature of its gene, thus making polymerase chain reaction (PCR)-based gene assembly methods challenging (Zhang et al., 2011). Cas also has an advantage in its ability to edit multiple genes simultaneously in multiplex editing (multiplexion) by supplying multiple gRNAs (McCarty et al., 2020).

Different natural and engineered variants of the CRISPR-Cas system have been discovered or developed in the years since its advent to optimize and customize this tool for different applications (Komor et al., 2017). Natural variants include Cas12a and Cas13. Like Cas9, Cas12a cleaves DNA to create DSB, but unlike Cas9, Cas12a remains partially bound to the target DNA whereupon it initiates nonspecific ssDNA cleavage (Paul and Montoya, 2020). Cas12a has been exploited and further engineered for higher efficiency plant genome engineering by virtue of its sticky-ended DSB and a cut-site outside of its recognition sequence which enables recutting unlike Cas9 (Merker et al., 2020). Cas13 (formerly C2c2) cleaves a ssRNA target rather than DNA (Abudayyeh et al., 2016) and has been used for RNA targeting, tracking, and editing in biotechnological, diagnostic, and therapeutic applications (Gupta et al., 2022; Pickar-Oliver and Gersbach, 2019). Engineered Cas variants include Cas9-nickase (“nCas”) which has been engineered to remove DNA-cutting activity from one of the two nuclease domains, leading to cutting of only one DNA strand and creating a ‘nick’ (Jinek et al., 2012). Paired nCas9 dimers have been employed to increase the specificity of Cas cutting because they require binding of two sgRNAs (Ran et al., 2013). Catalytically dead “dCas9” is another variant, engineered to retain DNA-sequence binding ability but devoid of cutting activity (Qi et al., 2013). dCas9 has been developed for transcriptional regulation, either through its ability to block RNA polymerase when bound at the target sequence (Qi et al., 2013) or through conjugating effector proteins to dCas9. Various effector proteins may be conjugated to dCas9 for varied applications, such as epigenetic regulation and investigation and manipulation of chromosomal organization (Adli, 2018).

In addition, Cas engineering efforts have focused on increasing nuclease specificity to reduce off-target cutting, essential for clean genome engineering (Bratovič et al., 2020). Conventional genome engineering relies on repair of the nuclease-mediated DSB either through accurate homologous recombination (HR) or error-prone non-homologous end joining (NHEJ) which leads to specific or random sequence alteration respectively at the target site. However, NHEJ nearly always accompanies HR and can lead to unwanted base insertions or deletions (indels). Recently, the ability to make small genetic changes without DSB induction — and thereby avoiding unwanted indel formation through erroneous NHEJ — has arrived in the form of base editing and prime editing, which use dCas9 or nCas (Anzalone et al., 2019; Komor et al., 2016). Although base editing is limited to certain single base conversions, prime editing delivers more versatility with longer sequence insertion and deletion possible, as well as all 12 base-to-base conversions. Epigenetic alterations can be equally achieved through e.g., an acetyltransferase-dCas9 fusion to modify gene regulation in a physiological way (Hilton et al., 2015; Engreitz et al., 2019). These functions are summarized in Figure 1.

Despite the overwhelming success of this technology in the decade since its description, challenges remain. Delivery of the large CRISPR-Cas9 ribonucleoprotein (RNP) complex to target cells is primary amongst them, particularly for human gene therapy (Liu et al., 2021). Potential immunogenicity of Cas proteins must also be addressed in therapeutic applications (Charlesworth et al., 2019). The field of DNA nanotechnology is well placed to address the Cas-gRNA (guide RNA) delivery challenge, and indeed has very recently been employed to do so (Lin-Shiao et al., 2022).

Throughout the past decades DNA nanotechnology has taken giant leaps toward its enabled state (Seeman and Sleiman, 2018; Nummelin et al., 2018). In a nutshell, the field has evolved from rather small and simple, yet elegant tile-based structures composed of a few DNA strands to more complex DNA structures with dozens of strands, such as DNA origami (Figure 1) and its variations (Dey et al., 2021). From these, a conventional DNA origami is assembled by folding a ∼7000-nt long single-stranded DNA scaffold into a defined shape by short staple strands that hybridize with multiple scaffold segments and thus form double-helical domains (Rothemund, 2006). It has become a major technique in the ever-expanding toolbox for sub-nanometer accurate DNA nanostructure design. Currently, automated design paradigms (Linko and Kostiainen, 2016; Huang et al., 2021a), meshed wireframe structures (Piskunen et al., 2020), ∼10^7^-nt-size discrete/finite structures (Wintersinger et al., 2022), and macroscopic lattices assembled from ∼10^12^ individual DNA origami components (Xin et al., 2021) are available. Moreover, inorganic nanostructure engineering (Heuer-Jungemann and Linko, 2021) and versatile chemical modifications for DNA (Madsen and Gothelf, 2019) are accessible for a variety of bioimplementations.

Here, we discuss programmable DNA nanostructure-based systems that could be integrated with CRISPR/Cas techniques to yield novel applications in bioengineering and therapeutics. First, we introduce the straightforward concepts of using DNA structures as carrier systems for CRISPR/Cas delivery. Then, we present more advanced recent approaches where DNA nanostructures have imbued CRISPR/Cas systems with additional functionality, or where, conversely, CRISPR/Cas has been implemented to functionalize DNA-based devices. Finally, we consider some future outlooks for the fusion of these two techniques. Although we focus on DNA-based applications, it is noteworthy to mention that RNA nanotechnology also allows for programmable strand-displacement schemes that can be used to conditionally activate CRISPR functions (Oesinghaus and Simmel, 2019; Lin et al., 2020). Besides these circuit-controlled systems, RNA can be used in constructing versatile nanoshapes that may have equally intriguing implementations in the CRISPR/Cas settings. Here, the interested reader is referred to the literature on rationally designed RNA nanostructures (Grabow and Jaeger, 2014; Liu et al., 2020a; Geary et al., 2021).

## Current challenges and the paradigm of integration

CRISPR/Cas has unprecedented potential across the breadth of biological sciences, biotechnology, and medicine and has already transformed research. To-date, there have been considerable advances in the application of this technology, including agricultural CRISPR/Cas-edited products brought to market (Waltz, 2018, 2022), bacterial metabolic engineering (Liu et al., 2020b) and, in medicine, gene editing-based approaches are already being used to develop novel therapies (Human Genome Editing (HGE) Registry, 2022). A key advantage of gene editing in therapeutic applications is the capacity for correcting the underlying mutations of severe genetic diseases rather than treating the symptoms. In addition, gene editing can cure dominant conditions that are harder to address through conventional gene therapy. These technologies are rapidly moving from model systems to clinical trials as illustrated by gene correction of sickle cell disease and β-thalassemia (Frangoul et al., 2021). Here, premade sgRNA/Cas9 complexes were electroporated into hematopoietic stem/progenitor cells *ex vivo* and the modified cells introduced into patients, resulting in the desired outcome of increased fetal hemoglobin expression. Despite the recent successes in the use of gene editing, this ground-breaking technology still requires refinement if it is to be more generally applicable.

The paradigm for gene editing is the ability to reach target cells *in vivo* without generating immunological reactions and to effect the desired changes with high efficiency and minimal off-target target effects such as ectopic Cas activity or integration of the engineering machinery. A critical aspect is the method of delivery, identified as a major bottleneck in the application of gene editing to many crop species (Atkins and Voytas, 2020). The presence of the plant cell wall provides a barrier to transgene delivery that can be overcome by biological transformation using *Agrobacterium* or biophysical methods including biolistic transformation. Homology dependent gene editing frequencies are typically low, with the exception of a recent report of tobacco transformation that displayed ∼10% successful gene targeting (Huang et al., 2021b; Puchta et al., 2022). The plant cell wall can be removed with enzymes to produce protoplasts that are amenable to electroporation or polyethylene glycol-mediated approaches and transformation with preassembled Cas-RNP complexes resulted in ∼5% gene targeting frequencies (Jiang et al., 2021b). An exciting new development demonstrated *in vivo* biolistic transformation of wheat with Cas-RNPs, targeting meristem cells and avoiding the requirement for plant regeneration (Kumagai et al., 2022). This technology has the potential to be widely applicable to crop species.

In human gene therapy, there are a number of delivery approaches that differ in efficacy and ease of use (Lino et al., 2018). Microinjection can be technically challenging, requiring suitable expertise and is typically used with oocytes and zygotes, whereas electroporation and lipofection are commercially available technologies that are suitable for DNA or Cas-RNPs. *In vivo* delivery methods include hydrodynamic gene transfer that works by injecting the cargo in a large volume into the bloodstream of animal models. This can result in trauma but also permeabilization of cells, allowing uptake of transgenes (Sayed et al., 2022). Viral vectors are often used as a trusted and well-established technology that provides a highly efficient method of gene delivery. However, adeno-associated viruses are limited in capacity to ∼5 kilobases, creating issues for packaging the gene editing machinery together with the homology-dependent repair template (Yin et al., 2017). The use of small Cas variants, including the newly discovered ancestral Cas-like nucleases associated with transposons (Altae-Tran et al., 2021; Karvelis et al., 2021) may help address this issue, although the restricted payload capacity remains a limitation for viral vectors. An additional drawback results from immunological reactions, in particular after repeated treatment, which can present a major impediment to viral-based therapies (Duncan, 2022). A third limitation to viral vectors is the lack of flexibility in terms of how the gene editing machinery can be delivered. This limits potential approaches to reduce off target effects. Viral delivery introduces the gene editing machinery to the cell, which can lead to extended periods of Cas expression and/or unwanted integration events.

Nanotechnology-based gene delivery has the potential to address many of the limitations of viral vectors. Nanotechnology is well established in nucleic acid-based therapies (Kulkarni et al., 2021) and has even greater potential when combined with gene editing technologies. Lipid-based nanoparticles have already demonstrated success in clinical trials, enabling *in vivo* gene editing in hepatocytes in six patients (Gillmore et al., 2021). Through optimized design, the gene editing machinery can be engineered to promote the desired outcome. For example, in mammalian cells, covalent linkage of the repair template to Cas9 increased homology-dependent repair-mediated gene editing at the site of an induced break (Savic et al., 2018). The possibilities of modifying the system are becoming increasingly extensive as nanotechnology advances. In particular, the inherent flexibility in the design of DNA nanostructures could be used for cell or tissue specific targeting and fine-tuning of when, where, and how long the gene editing machinery is expressed. However, stability of the designer DNA nanostructures in physiological conditions still remains a challenge (Ramakrishnan et al., 2018; Bila et al., 2019).

## DNA-enabled delivery of CRISPR/Cas systems

As the modular DNA nanostructures are inherently biocompatible and possess exceptional addressability (Funke and Dietz, 2016), their use in biomedical settings is coming increasingly into view (Hu et al., 2019; Keller and Linko, 2020; Jiang et al., 2021a). DNA nanostructures have been prominently investigated as programmable drug delivery platforms that enable protection, targeting, and controlled release of cargo (Linko et al., 2015; Surana et al., 2015). The delivery of gene editing tools using DNA nanotechnology has also lately garnered attention from various research groups. Attempts have been made to functionalize other carriers with DNA or even building the carriers entirely from DNA components. Thus, sophisticated carrier systems have been introduced rather concurrently in recent years.

**Figure 2:**
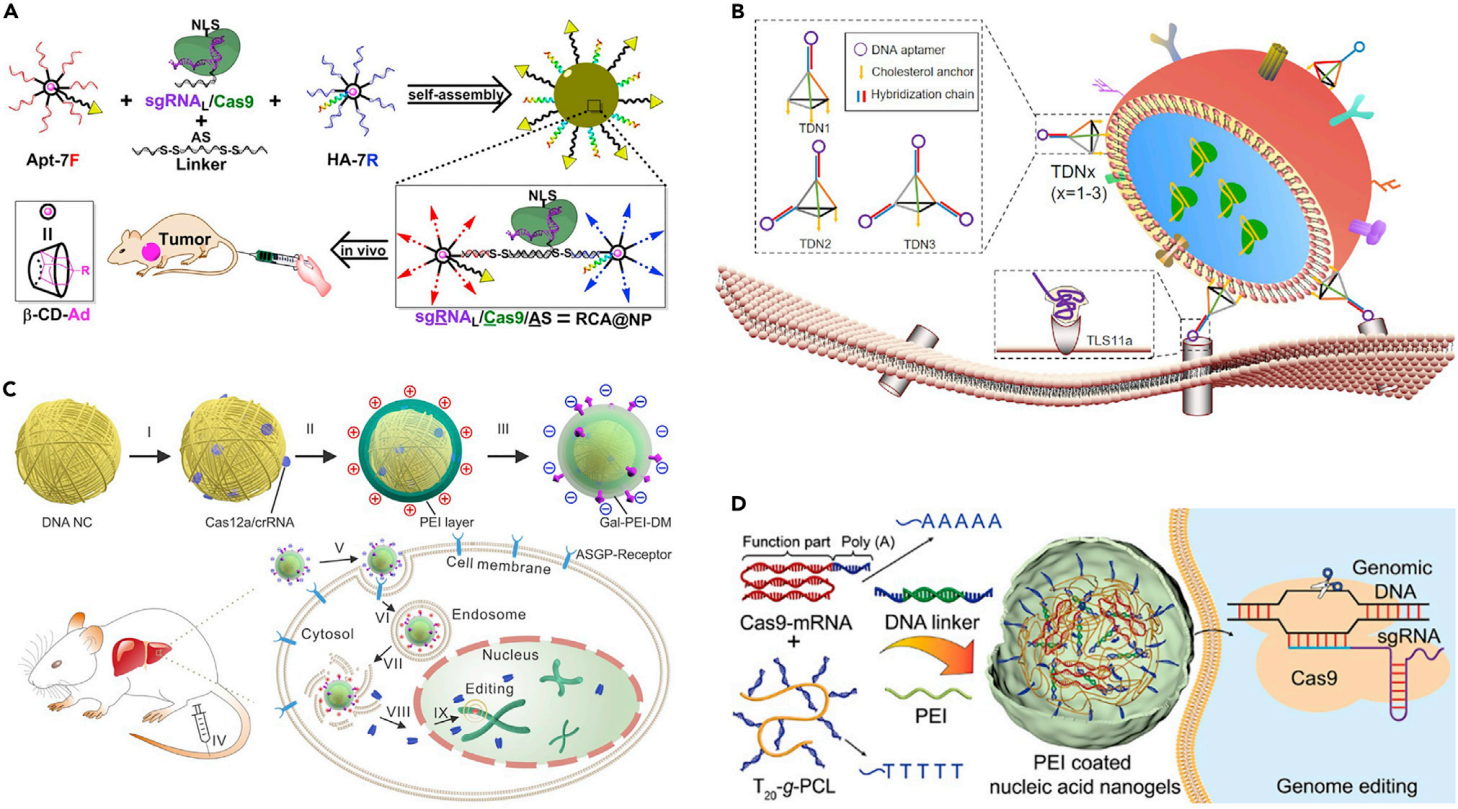
Delivery systems through CRISPR/Cas-DNA nanostructure fusion (A) β-cyclodextrin cores decorated with linear DNA branches. (B) Tetrahedral DNA nanostructures with vesicle-binding cholesterol linkers and cell-targeting aptamers. (C) Polymer-coated charge-reversible DNA nanoclews. (D) Polymer-coated PCL nanogels cross-linked with DNA linkers. (A) adapted with permission from (Liu et al., 2019); Copyright (2019) American Chemical Society. (B) adapted with permission from (Zhuang et al., 2020); Published (2020) by Oxford Academic Press. (C) adapted with permission from (Sun et al., 2020); Published (2020) by The American Association for the Advancement of Science. (D) adapted with permission from (Huang et al., 2020); Copyright (2020) American Chemical Society.

In one of the simplest of these approaches, linear DNA was employed in the carrier platform (Liu et al., 2019), in which seven ssDNA arms were covalently linked to azide-modified β-cyclodextrin cores. These branched DNA structures (dubbed 7F and 7R) could be mixed with linker and sgRNA to assemble them into a sgRNA/Cas9/antisense-nanoparticle (RCA@NP) (Figure 2A), where the sgRNA/Cas9 still retained its gene editing efficacy. An aptamer modification enhanced the targeted delivery of their RCA@NP complexes into human cancer cells and release of cargo was demonstrated with digestion of the carrier by glutathione and RNase H. Zhuang et al. (2020), meanwhile, showed how even an already existing carrier could be simply modified by well-known and modular DNA nanostructures. They functionalized extracellular vesicles (EVs) with valency-controlled tetrahedral DNA nanostructures (TDNs) that contained cholesterol anchors for binding with the vesicle surface and DNA aptamers for cell targeting (Figure 2B). The modular TDNs facilitated tumor-specific aiming of the EVs and thereby also targeted delivery of CRISPR/Cas9 loaded inside the carriers.

Somewhat earlier, in a combination of similar ideas as those later employed by Liu et al. (2019) and Zhuang et al. (2020), Sun et al. (2015) envisioned a sgRNA/Cas9-loaded DNA nanoclew. The nanoclew consisted of a yarn-like long DNA strand that wrapped into a spherical shape during rolling circle amplification (RCA). The repeating strand sequence was chosen to be partially complementary to that of the used sgRNA to enable loading of the clews with sgRNA/Cas9 complexes. By coating the loaded clews with a cationic polymer polyethyleneimine (PEI), they were able to ease the endosomal escape of the carriers inside cells. The same group later expanded upon the design of the DNA nanoclews (Sun et al., 2020). They further functionalized the PEI-coated carriers with an additional charge reversal polymer layer of galactose-PEI-2,3-dimethylmaleic anhydride (Gal-PEI-DM), which effectively reversed the carrier’s charge in response to an environmental pH change from physiological to acidic (Figure 2C). In this work, Sun et al. used the clews for the successful *in vitro* and *in vivo* delivery of a Cas12a/CRISPR RNA (crRNA) RNP system that aimed to reduce cholesterol serum levels in mice.

Another comparable delivery strategy was conceived by Ding et al. (2019) who created a DNA-based nanogel system instead of a nanoclew. The nanogel carriers were created by first loading DNA-grafted polycaprolactone brushes (DNA-g-PCL) with sgRNA/Cas9 complexes and then crosslinking via hybridization with DNA linkers. The non-cationic nanogel shielded the sgRNA/Cas9 complexes packed inside of it against nuclease digestion and facilitated gradual release as the gel was digested. In a subsequent work, it was shown that these gels could also be coated with PEI (Huang et al., 2020) to improve endosomal escape like in the previous nanoclew studies (Figure 2D). Their functionality was demonstrated by loading and delivering Cas9 protein-encoded mRNA (Cas9-mRNA) and enhanced green fluorescence protein-encoded mRNA (EGFP-mRNA) cargo. The mRNA was bound via hybridization of their poly-A tails to the poly-T segments of the nanogel framework (poly-T_20_-grafted polycaprolactone (T_20_-g-PCL)).

Finally, Li et al. (2022) recently reported a proton-activated co-delivery system based on ultralong ssDNA including sgRNA recognition sites for sgRNA/Cas9 attachment, DNAzyme sequences, and *Hha*I enzyme cleavage sites. The DNA strands were compressed into nanoparticles using DNAzyme cofactor Mn^2+^, and the particles were further equipped with acid-degradable polymer-coated *Hha*I enzymes. The acidic environment in lysosomes could then trigger polymer decomposition allowing Hhal to cut off the cleavage sites and release both sgRNA/Cas9 and DNAzymes for gene expression regulation in breast cancer cells.

## Designer DNA origami with CRISPR/Cas: Delivery and beyond

**Figure 3:**
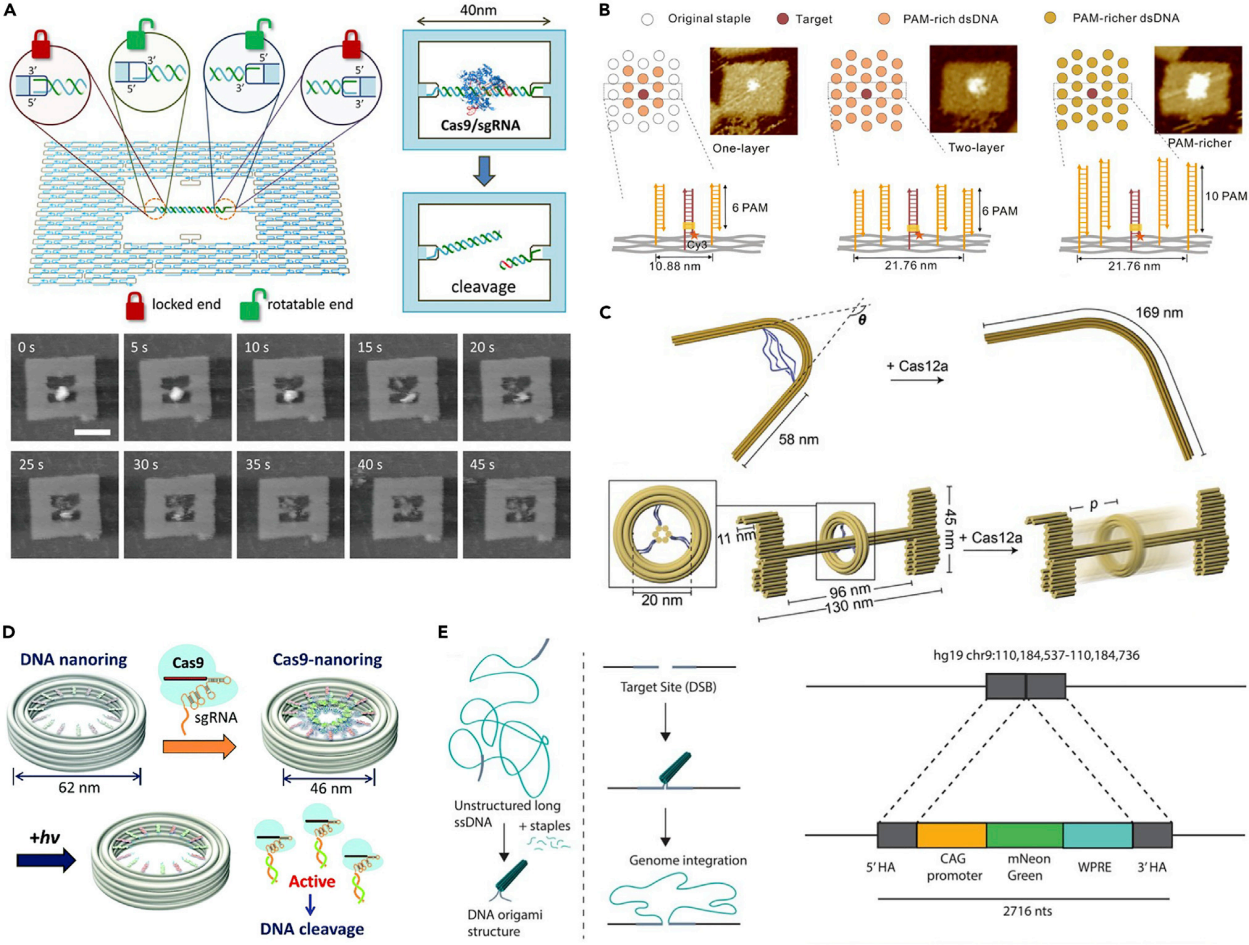
Advanced applications of CRISPR/Cas-DNA origami nanosystems (A) DNA origami frame for high-speed AFM analysis of Cas cleavage of DNA targets. (B) PAM antennas on DNA origami. (C) Post-processing of DNA origami with CRISPR/Cas. (D) Light-controlled DNA origami-CRISPR/Cas system. (E) Delivery of genes packed into a DNA origami platform. (A) adapted with permission from (Räz et al., 2016); Copyright (2016) American Chemical Society. (B) adapted with permission from (Wang et al., 2020a); Published (2020) by The American Association for the Advancement of Science. (C) adapted with permission from (Xiong et al., 2020); Copyright (2019) John Wiley & Sons. (D) adapted with permission from (Abe et al., 2021); Copyright (2021) by Royal Society of Chemistry. (E) adapted with permission from (Lin-Shiao et al., 2022); Published (2022) Oxford Academic Press.

In addition to the more straightforward delivery systems, the combination of DNA nanostructures and CRISPR/Cas has also yielded other kinds of intriguing applications, such as studying the fundamental interactions between CRISPR/Cas and DNA. To this end, Räz et al. (2016) designed a tile-like DNA origami frame for systematically studying the Cas cleavage of DNA through atomic force microscopy (AFM). The hollow of the frame (Figure 3A, top panel) contained a binding site for suspending dsDNA sequences from two opposing points in either rotatable or constrained manners. This setup allowed the authors to study how Cas is able to bind to and cleave relaxed and restrained targets in real time using a high-speed AFM (Figure 3A, bottom panel).

In a comparable study, Wang et al. (2020a) utilized the submolecular-scale positioning power of DNA origami to confine PAM antennas to the vicinity of a target DNA (Figure 3B). This enabled investigation of the dynamics of sgRNA/Cas9 binding and cleaving efficiency in the presence and absence of the PAM antennas. By following the binding of sgRNA/dCas9 onto the DNA origami via AFM imaging, they observed that PAM antennas attract Cas9 molecules and thus promote the cleavage of target DNA in their proximity. Moreover, they noted an increased density of the antennas could further increase the cleavage efficiencies of closely located targets.

CRISPR/Cas has also enabled the post-processing of DNA origami into otherwise impossible configurations and for inducing dynamic conformational changes as was demonstrated by Xiong et al. (2020). They employed CRISPR-Cas12a to systematically reconfigure ready-made DNA origami structures from predefined cleavage points. To achieve this, they used the Cas12a to nonspecifically cleave single-stranded features in DNA origami, either to simply remove unfolded scaffold segments or, more intriguingly, to release moving parts or tension-loaded shapes in more complex structures (Figure 3C).

In a twist to the previously introduced delivery applications, Abe et al. (2021) presented a DNA origami nanoring that could be employed for the triggered release of Cas9. The authors anchored Cas9 onto the inner surface of a ring-shaped DNA origami through photoresponsive linkers and subsequently released the Cas9 in a controlled manner upon light irradiation of the structures (Figure 3D). Their design demonstrates how the activity of Cas9 can in principle be completely suppressed in a carrier system until a trigger is introduced.

In a very recent accomplishment, Lin-Shiao et al. (2022) designed a DNA nanostructure carrier approach for more efficient transport of even longer gene sequences into human cells. Rather notably, they exploited DNA origami folding mechanisms to pack an entire gene-length ssDNA sequence into a compact structure for cellular delivery. In their design a truncated Cas9 target sequence was attached to both ends of a linear scaffold (the delivered gene sequence), and the addition of synthetic staple strands was used to create a complete DNA origami shape (Figure 3E). This process resulted in a compact, predefined 18-helix DNA origami tube where both Cas9 targets protruded from one end of the origami. This property was employed to effectively modulate the end-to-end distance of the Cas9 targets from long (>100 nm) to short ones (<40 nm). The nanostructures were also decorated with binding sites for Cas9 RNPs to improve their shuttling to cell nuclei. The thus structured genes displayed improved delivery and genomic integration in comparison to unstructured genes. In the study, Lin-Shiao et al. demonstrated the delivery of their DNA nanostructures to cell nuclei via electroporation and also by using Cas9 virus-like particles (VLPs).

## Outlook

In this article, we have introduced a number of recently developed techniques for merging the realms of CRISPR/Cas systems and rationally designed DNA nanostructures (summarized in Table 1). As the integration of CRISPR/Cas systems and programmable/functional DNA nanostructures is in its infancy, several directions should be further explored.

**Table 1:** Summary of the selected DNA platforms, their types of action, and promoted applications

Classification/DNA platform type	Type of action	Application	References
**Delivery**			

Branched ssDNA structures	Linking of sgRNA to the structures to form sgRNA/Cas9/antisense particles	Aptamer targeted delivery and release of sgRNA/Cas9 upon enzymatic digestion	Liu et al. (2019)
Valency-controlled tetrahedral DNA nanostructures (TDNs)	Anchoring of aptamer-equipped TDNs to EVs	Tumor-specific EV targeting	Zhuang et al. (2020)
PEI-coated DNA nanoclew	Repeating strand sequence complementary to sgRNA	sgRNA/Cas9 delivery and aided endosomal escape	Sun et al. (2015)
Gal-PEI-DM coated DNA nanoclew	Addition of charge-reversal polymer coating to DNA nanoclew	Charge-reversal of the carrier in response to pH changes	Sun et al. (2020)
DNA nanogel	Crosslinking of sgRNA/Cas9 loaded DNA-grafted PCL brushes	Gradual release upon digestion, aided endosomal escape (with PEI coating)	Ding et al. (2019); Huang et al. (2020)
Ultralong ssDNA encoded with multiple functional sites	Proton triggered release of Hhal enzyme causes cleavage of carrier DNA at encoded cleavage sites	Co-delivery of sgRNA/Cas9 and DNAzyme inside lysosomes	Li et al. (2022)
Gene-based DNA origami	Folding of gene-length ssDNA into DNA origami	Co-delivery of entire genes and sgRNA/Cas9	Lin-Shiao et al. (2022)

**Other functions**			

Tile-like DNA origami frame	Binding sites in the hollow of the frame allow controlled suspension of target from two points	Studying the Cas cleavage of relaxed and restrained dsDNA targets in real time with high-speed AFM	Räz et al. (2016)
DNA origami tile decorated with PAM antennas	Controlled positioning and confinement of PAM antennas near sgRNA targets	Studying of sgRNA/Cas9 binding and cleaving dynamics	Wang et al. (2020a)
DNA origami with ssDNA cleavage sites	Cleavage of ssDNA features with Cas12a	Post-processing and release of moving or tension-loaded DNA origami structures	Xiong et al. (2020)
Photoresponsive DNA origami nanoring	Anchoring of Cas9 to DNA origami with photoresponsive linkers	Remote-triggered release of Cas9	Abe et al. (2021)

### *In vivo* stability and functionality of hybrid nanostructures

One of the obvious challenges is the translation of the *in vitro* applications to physiological environments. Although CRISPR/Cas systems have been harnessed to achieve ultrasensitive detection of nucleic acids, which are described in a recent review article by Wang et al. (2021), nucleic acid nanostructures need to display several qualities to realize their full potential in *in vivo* applications. More specifically, it is necessary to achieve both high stability and cell-specific reactivity within biological systems and nanostructure compactness for reaching the nucleus, whereas simultaneously ensuring negligible immunological reactions at the organism level. Furthermore, the nanostructures should also be capable of input-specific release of molecular cargo.

Other biophysical insights can underpin new DNA architecture design. Harnessing the function of sequence-independent, house-keeping enzymes with nucleolytic function available in the cell environment offers an alternative approach to develop the multistage cargo-delivery architecture described previously. For instance, RNase H digests the RNA strand of hybrid DNA:RNA duplexes and has been already applied to integrate CRISPR/Cas systems and DNA nanostructures (Zhuang et al., 2020). However, only very recent studies have systematically elucidated its reaction with synthetic, linear DNA/RNA substrates. Studying the hepatitis B virus RNase H, Villa et al. (2016) have demonstrated that its sequence-nonspecific, distributive, and endonucleolytic activity requires DNA/RNA duplex stretches of at least 14 nt, and is silenced by the presence of a stem-loop structure in either one of the two strands, or a gap in the DNA strand.

In a very recent breakthrough article, Lee et al. (2022) discovered the dual functionality of the *E. coli* RNase H, whose function crucially depends on symmetry of the DNA overhang. Using surface-bound DNA:RNA chimeric probes and Förster resonance energy transfer (FRET) analysis, the authors uncovered that with 3′ ssDNA overhang, RNase H works as a processive exoribonuclease that continuously degrades RNA from 5′ to 3′. Comprehensively, these results suggest the possibility of triggering the RNase H-mediated disassembly of nanostructure components containing DNA:RNA hybrid duplexes, by varying/modulating the RNase H binding site within a hybrid DNA:RNA nanostructure.

### Emergent allosteric properties of DNA nanostructures

This aforementioned approach would simplify nanostructure design, chemical composition, and therefore, synthesis. In contrast, however, it would require overcoming other limitations such as the steric inhibition of enzymatic reactions within DNA nanostructures (Ramakrishnan et al., 2019; Ijäs et al., 2021; Xin et al., 2022). Stopar et al. (2018) showed that restriction enzyme cleavage of a “sharp triangle” DNA origami nanostructure exhibits a digital on/off behavior, in that for each site in the triangle, the endonuclease action is either highly efficient or fully inhibited. Moreover, for a specific restriction enzyme (*Hha*I), introduction of structural defects in the triangle (lacking only four staples) activates otherwise unreactive sites, with a site-to-defect distance of nearly 50 nm. The results – fully consistent with the behavior of ten restriction enzymes on the same DNA nanostructure – show that nucleolytic action on a DNA origami can be regulated in a digital fashion through local structural control of DNA-enzyme recognition. In particular, the presence or absence of a DNA nick can allosterically control the reactivity of an adjacent restriction site.

Despite the work proposing an empirical model accounting for the mechanical rigidity around restriction sites, accurate interpretation of the results would require computational modeling to describe the details of protein-DNA nanostructure interactions. For example, Suma et al. (2020) developed a computational approach, based on the coarse-grained model oxDNA (Sengar et al., 2021) to parametrize the local accessibility of the DNA triangle to *Hin*P1I endonuclease (an isoschizomer of *Hha*I), obtaining good agreement with the experimental data. According to this study, the endonuclease action was regulated by both global and local mechanical properties of a DNA origami triangle linked to the existence of metastable conformations that significantly change in nanostructure variants containing even small defects because of the increase of global fluctuation. These results introduce the possibility of varying the allosteric properties of DNA nanostructures to regulate biomolecular recognition and reactions, which is a new concept in DNA nanotechnology. Pursuing this direction will therefore require further investigations to identify structural determinants and antideterminants of Cas enzymes under nanoscale confinement.

### Enhancing CRISPR/Cas systems by compacting DNA into nanostructures

Integrating all these features challenges nucleic acid nanostructure design, whereas multistage robotic cargo release approaches could offer more feasible solutions (DeLuca et al., 2020; Nummelin et al., 2020). For instance, CRISPR/Cas systems could be designed to process intermediate, large nucleic acid-based nanostructure carriers with smaller, functional nanostructure components, which could subsequently release genetic material inside the nucleus triggering gene editing. Using CRISPR/Cas systems to transform a DNA nanostructure rather than relying on protein-protein or other protein-nucleic acid interactions trigger cargo release. Moreover, the advantage of using biocompatible coatings may help to fulfill this strategy. DNA platforms could be further functionalized with proteins such as BSA, designer peptoids, or polymers like oligolysines which have been shown to enhance stability, transfection, and immunocompatibility (Auvinen et al., 2017; Wang et al., 2020b; Anastassacos et al., 2020).

Another direction to integrate CRISPR/Cas systems and programmable DNA nanostructures has been recently signposted by the disruptive approach (Lin-Shiao et al., 2022). Their proposal opens the door to DNA origami nanostructures that are designed to enhance or suppress DNA integration (depending on the application, see the previous sections), or even direct homologous recombination.

Finally, in the shorter term, building on genomic integration of a scaffold-like transgene by involving transgene-dependent staples, the next step could be developing a universal strategy for transgene incorporation by freeing (or minimizing) DNA staple design, based on a programmable, transgene nanocarrier that remains inert during the DNA integration process.
